# Effect of Overeating Dietary Protein at Different Levels on Circulating Lipids and Liver Lipid: The PROOF Study

**DOI:** 10.3390/nu12123801

**Published:** 2020-12-11

**Authors:** George A. Bray, Leanne M. Redman, Jennifer Rood, Lilian de Jonge, Steven R. Smith

**Affiliations:** Pennington Biomedical Research Center-LSU, Baton Rouge, LA 70808, USA; leanne.redman@pbrc.edu (L.M.R.); jennifer.rood@pbrc.edu (J.R.); edejonge@gmu.edu (L.d.J.); steven.r.smith@adventhealth.com (S.R.S.)

**Keywords:** dietary protein, protein overfeeding study (PROOF), plasma lipids, liver fat

## Abstract

Background: During overeating, a low protein diet slowed the rate of weight gain and increased the energy cost of the added weight, suggesting that low protein diets reduced energy efficiency. The Protein Overfeeding (PROOF) study explored the metabolic changes to low and high protein diets, and this sub-study examined the changes in body composition and blood lipids when eating high and low protein diets during overeating. Methods: Twenty-three healthy volunteers (M = 14; F = 9) participated in an 8-week, parallel arm study where they were overfed by ~40% with diets containing 5% (LPD = low protein diet), 15% (NPD = normal protein diet), or 25% (HPD = high protein diet) protein. Dual energy X-ray absorptiometry (DXA) and computer tomography (CT) were used to quantify whole body and abdominal fat and intrahepatic lipid, respectively. Metabolites were measured by standard methods. Results: Protein intake and fat intake were inversely related since carbohydrate intake was fixed. Although overeating the LPD diet was associated with a significant increase in high density lipoprotein (HDL)-cholesterol (*p* < 0.001) and free fatty acids (*p* = 0.034), and a significant decrease in fat free mass (*p* < 0.0001) and liver density (*p* = 0.038), statistical models showed that dietary protein was the main contributor to changes in fat free mass (*p* = 0.0040), whereas dietary fat was the major predictor of changes in HDL-cholesterol (*p* = 0.014), free fatty acids (*p* = 0.0016), and liver fat (*p* = 0.0007). Conclusions: During 8 weeks of overeating, the level of dietary protein intake was positively related to the change in fat free mass, but not to the change in HDL-cholesterol, free fatty acids, and liver fat which were, in contrast, related to the intake of dietary fat.

## 1. Introduction

Obesity is a major world-wide health problem [1,2], and understanding how it develops and what can be done to prevent and treat it are thus important challenges for the public and health professionals [3]. Various physical, metabolic, and endocrine changes have been described in spontaneous obesity in man [4], and overfeeding has been one strategy used to probe the complex regulation of body weight in human beings [5]. In 1999, Stock reviewed studies of overeating and showed that the amount of protein in the diet had a significant effect on the amount of weight gained, and the energy cost of this weight gain [6]. Both low protein diets with protein levels between 2 and 8% of energy as well as high protein diets with 20% of energy from protein were associated with increased energy cost of weight gain. In the same year, Dulloo and Jacquet proposed that low protein diets could be an effective strategy to unmask susceptibility to obesity [7].

With this as a background, we designed the Protein Overfeeding (PROOF) study to examine the effects of low and high protein diets in comparison to a normal protein diet [8]. The increase in body fat with overeating was directly related to the excess energy intake, and independent of protein intake. On the other hand, the increase in fat free mass was significantly reduced in the low protein diet compared to the other 2 diets [8]. The low level of protein might well be expected to alter metabolic responses in systems requiring synthesis of proteins. The level of overfed dietary protein effected the circulating levels of both essential and non-essential amino acids [9]. Six of the essential amino acids increased as dietary protein increased, but in contrast, lysine, an essential amino acid, was significantly higher in those eating the low protein diet (*p* = 0.040). Dietary protein was also a major factor in the changes in levels of fatty acyl carnitines, particularly the low protein diet [10]. These effects of ingesting a low protein diet during overeating prompted the current investigation which asked whether different levels of dietary protein intake would significantly influence distribution of body fat, the levels of circulating lipids and liver fat, since none of the 3 previous papers on low protein diets and overfeeding included these data [6,11,12].

## 2. Methods and Materials

### 2.1. Participants

In this parallel arm study, 14 men and 9 women between the ages of 18 and 35 years with a BMI between 19.7 and 29.6 kg/m^2^ who led a sedentary lifestyle were recruited and randomly assigned to one of three diets with either 5% (LPD = low protein diet), 15% (NPD = normal protein diet), or 25% (HPD = high protein diet) protein. The protocol was approved by the PBRC Institutional Review Board (IRB # PBRC25007) and registered at Clinicaltrials.gov (NCT00565149). Each individual signed an informed consent after receiving a full explanation of risks and benefits.

### 2.2. Protocol

Details and many results from this randomized, parallel-arm in-patient study have been previously published [8,9,10,11,12,13,14,15,16]. Briefly, participants were admitted to the metabolic ward where energy requirements for weight maintenance were established over the first 3 weeks. They were then randomized to one of 3 diets and began 8 weeks of overeating while remaining in-patients on the metabolic unit.

### 2.3. Diets

The three experimental diets were designed using the MENu 6.0 (Nutrient Data Base, Pennington Center, Baton Rouge, LA, USA) and provided either 5%, (LPD) 15% (NPD) or 25% (HPD) of energy from protein (Table 1). Volunteers were overfed by 38–42% above baseline energy requirements with food provided in 5-day rotating menus [8]. Analysis of food composites from each diet was performed at the end of the study by Covance Laboratories (Madison, WI) and showed that protein intake was 90 ± 16 g/day at baseline, and 47 ± 4.7, 140 ± 29, and 228 ± 48 g/day in the LPD, NPD, and HPD diets. This translated into 1.83 ± 0.98 g/kg/day of protein at baseline and 0.68 ± 0.069, 1.80 ± 0.25, and 3.01 ± 0.29 g/kg/day during overeating in the LPD, NPD, and HPD groups, respectively [15]. Carbohydrate was kept constant across the 3 diets and therefore the difference in energy was achieved by increasing dietary fat (Table 1). The baseline diet contained 60%en as carbohydrate. During overeating, this declined to 42–43% of calories. Fat intake was thus 49%en in the LPD, 42%en in the NPD, and 30%en in the HPD. Intake of saturated fat, mono- and polyunsaturated fat (MUFA and PUFA) was determined using the MENu 6.0 database as well as by direct measurement on pooled samples of each diet. These data are shown in Table 1.

### 2.4. Body Composition

A 3-compartment model of body composition was obtained using dual-energy X-ray absorptiometry (DXA, Hologics QDR 4500A, Marlborough, MA, USA). Scans were analyzed with the V11.1 QDR software for Windows [14].

### 2.5. Measurement of Adipose Tissue Volumes and Liver Fat

Abdominal adipose tissue was measured by computed tomography using a local hospital General Electric Computed Tomography machine (GE Milwaukee, Brookfield, WI, USA). Based on the scout film identifying the L4–L5 inter-vertebral disc, 8 contiguous images were obtained every 5 cm with 5 slices above and 2 below. Images were analyzed with the AnalyzePC^TM^ software and quantified as intra-abdominal visceral, subcutaneous-deep, and subcutaneous-superficial using a triangulation method [17]. Liver fat was estimated from the changes in liver density on the CT scans.

### 2.6. Adipose Tissue Biopsy for Fat Cell Size

Approximately 500 mg of adipose tissue was removed from a 1 cm incision on the abdomen, 5 cm from the umbilicus. Approximately 50 mg of tissue was placed in osmium tetroxide for the determination of fat cell size as previously described [18].

### 2.7. Laboratory Measurements

Metabolites were measured in blood specimens collected in the morning following a 12 h overnight fast. Glucose was measured using a glucose oxidase electrode, total cholesterol, Low density lipoprotein (LDL)-cholesterol, high density lipoprotein (HDL)-cholesterol were measured with a Beckman Coulter DXC 600 Pro, (Brea, CA, USA). Free fatty acids were measured with a high sensitivity kit (Wako Kit. Wako Diagnostics, Mountain View, CA, USA). Leptin was measured by immunoassay.

## 3. Statistical Analysis

Protein diets were randomly assigned. Baseline data are expressed as mean ± SD and change from baseline as mean ± SE. Changes related to the assigned protein diet were the primary end point for this study and were evaluated by analysis of variance (ANOVA) with the differences between groups being assessed with the Tukey–Kramer test. A linear model was used to assess the effects of dietary fat components and overfed protein on fat free mass, HDL-cholesterol, free fatty acids, and liver fat. The alpha was set at *p* < 0.05, and no corrections were made for multiple comparisons since this was a post-hoc analysis. Analyses were done using the JMP-7 statistical package (SAS, Cary, NC, USA).

## 4. Results

### 4.1. Associations with Dietary Protein Assignment

The effects of overeating protein at the three assigned levels on the metabolic parameters are summarized in Table 2. This table shows the data analyzed in 3 ways: First, the overall effect of overeating is shown in the right hand column; second, the change from baseline to 8 weeks for the LPD, NPD, and HPD groups was evaluated with statistical differences identified as superscripts in the change columns; and third, differences between diet assignments was evaluated using analysis of variance.

The effect of overeating, independent of diet assignment, was evaluated by pooling individual data from all 3 levels of dietary protein. All of the measures in Table 2 in the right-hand “Overall” column, except liver density, change in LDL/HDL and plasma free fatty acids changed significantly (*p* < 0.05) with overeating.

The changes from baseline to 8 weeks of overeating for each of the 3 diets were examined next. Body weight, (*p* < 0.001) body fat (*p* < 0.01), deep (*p* < 0.05) and superficial (*p* < 0.001) adipose tissue, and leptin (*p* < 0.01) increased significantly in all 3 diet groups as indicated by the superscripts in the change columns for LPD, NPD, and HPD in Table 2. Additional increases were seen in the LPD group for cholesterol (*p* < 0.01), LDL cholesterol (*p* < 0.01), and glucose (*p* < 0.01). Liver density decreased (*p* < 0.05), (i.e., liver fat increased). Fat free mass was unchanged in the LPD group, but significantly increased in the NPD (*p* < 0.01) and HPD (*p* < 0.001) groups (Table 2).

**Figure 1:**
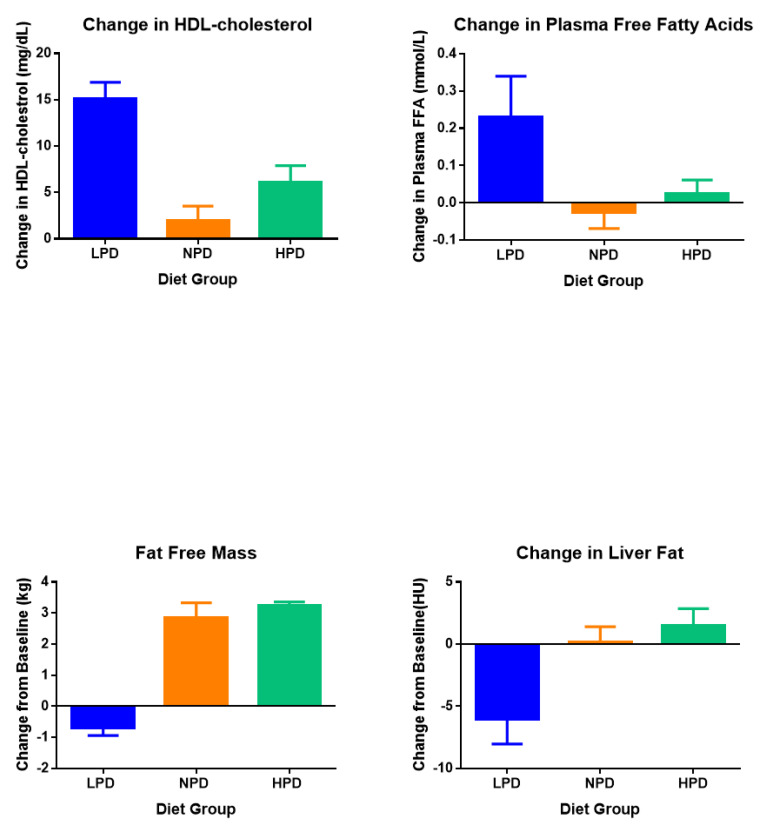
Changes in HDL-cholesterol, plasma free fatty acids, fat free mass, and liver density during overfeeding with diets containing different levels of dietary protein. (Tukey–Kramer analysis shows that for HDL-cholesterol: LPD > NPD (*p* < 0.0001) and HPD (*p* = 0.0072); For plasma free fatty acids: LPD > NPD (*p* = 0.034) NPD = HPD; for liver density: LPD > NPD (*p* = 0.038) and HPD (*p* = 0.012); for fat free mass: LDP < NPD and HPD (*p* < 0.0001). There were not significant differences between the NPD and HPD groups in any of the measurements.

The difference between the LPD, NPD, and HPD diets, assessed by analysis of variance (ANOVA) is shown in the second column from the right in Table 2 and is depicted in Figure 1. The low protein diet (LPD) was associated with significantly higher values for HDL-cholesterol than the NPD (*p* < 0.0001) or the HPD (*p* = 0.0072). Plasma free fatty acids were also significantly higher in the LPD group than the NPD group (*p* = 0.034), but not the HPD group which were not significantly different from each other. In contrast, liver fat measured as liver density and fat free mass were significantly reduced in the LPD group compared to the HPD (*p* = 0.012) and the NPD (*p* = 0.038) group, with no difference between the NPD and HPD groups. For the remainder of this paper, we will focus on the changes in fat free mass, HDL-cholesterol, liver fat, and free fatty acids (FFA).

### 4.2. Effects of Dietary Fat on HDL-Cholesterol, Total Cholesterol, Free Fatty Acids, and Liver Fat

**Figure 2:**
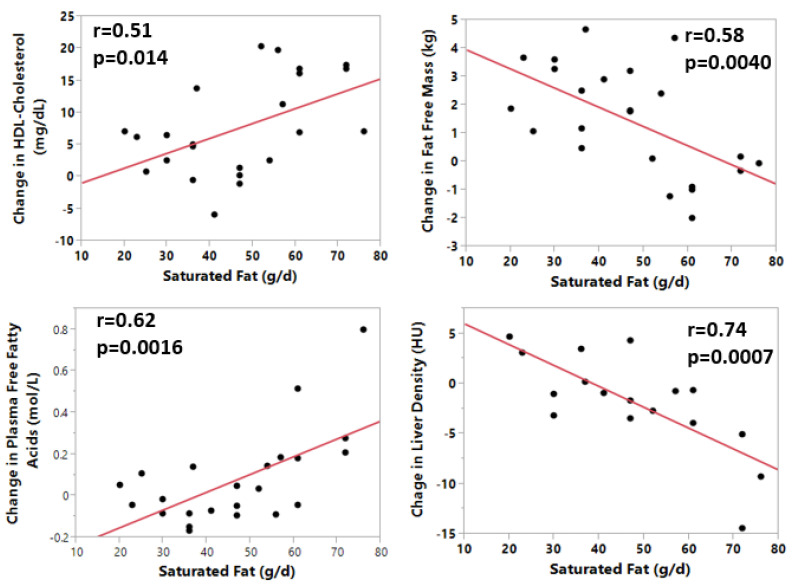
Relation of saturated fat intake to change in HDL-cholesterol, free fatty acids, fat-free mass, and liver fat during overeating.

Dietary fat intake was reciprocally related to protein intake and increased with body weight. Table 3 shows the correlation coefficients and statistical significance for the relationship of total fat, saturated fat, monounsaturated fatty acids (MUFA), and polyunsaturated fatty acids (PUFA) to the changes in the metabolic variables in this study. Total fat was positively related to body fat (*r* = 0.49, *p* = 0.033) and visceral adipose tissue (VAT), (*r* = 0.49, *p* = 0.038). Saturated fat was positively related to the rise in HDL-cholesterol (*r* = 0.51, *p* = 0.014) and inversely related to fat free mass (*r* = −0.58, *p* = 0.0093). Total fat (*r* = −0.64, *p* = 0.0046), saturated fat (*r* = −0.74, *p* = 0.0008), MUFA (*r* = −0.54, *p* = 0.013), and PUFA (*r* = −0.48, *p* = 0.045) were all negatively related to liver density (i.e., positively related to liver fat). Total fat (*r* = 0.056, *p* = 0.0056), saturated fat (*r* = 0.62, *p* = 0.0016), and MUFA (*r* = 0.48, *p* = 0.018) were also positively related to the changes in plasma free fatty acids (Table 3). Saturated fat had the strongest statistical relationship to changes in fat free mass, HDL-cholesterol, free fatty acids, and liver fat which are depicted in Figure 2. Both HDL-cholesterol (*r* = 0.51, *p* = 0.014) and free fatty acids (*r* = 0.61, *p* = 0.0016) were positively and significantly related to saturated fat, whereas fat-free mass (*r* = 0.58, *p* = 0.0040) and liver density (the inverse of liver fat) (*r* = −0.74, *p* = 0.0007) were significantly and negatively related to dietary saturated fat.

A statistical model was used to evaluate the relative contribution of overfed protein and dietary fat to the changes in fat free mass, HDL-cholesterol, free fatty acids, and liver fat. For the change in HDL-cholesterol, saturated fat was the most significant component in the model (*p* < 0.005). Saturated fat was again by far the strongest predictor of the change in plasma free fatty acids (*p* = 0.0007). A similar result was seen for liver density (liver fat) where saturated fat was the strongest predictor (*p* = 0.0012). In contrast, overfed protein was the major contributor to the change in fat free mass (*p* < 0.0001). Neither monounsaturated fats nor polyunsaturated fats contributed to these changes.

## 5. Discussion

In this study, participants received a constant daily intake of carbohydrate and were randomly assigned to one of 3 levels of dietary protein (5% = LPD; 15% = NPD; or 25% HPD) with the difference being made up by dietary fat. The analysis plan had as its primary end-point, changes resulting from the effects of dietary protein. Analysis of dietary fat, as done in this study, was thus an unplanned, post-hoc analysis. The low protein diet (5% energy from protein) was associated with a significant increase in HDL-cholesterol, plasma free fatty acids, and liver fat and a reduction in fat free mass (FFM) during 8 weeks of overeating in healthy men (*n* = 14) and women (*n* = 9). However, when the effect of dietary fat and dietary protein were examined together in a statistical model, it was dietary fat, but not dietary protein, that was associated with the changes in HDL-cholesterol, free fatty acids, and liver fat. Dietary protein was only associated with the change in fat free mass.

### 5.1. Overall Effects of Overfeeding

Overeating produces a wide variety of metabolic changes [4,5]. In this study, we examined these changes from baseline to 8 weeks of overeating, independent of diet, by pooling data from all 3 dietary groups. Using this pooled data, triglycerides decreased significantly and all other variables increased significantly except for liver fat, free fatty acids, and the LDL/HDL ratio. In response to overeating, changes in lipids and lipoproteins have varied with the length of the study, as well as the type of diet that was provided [5]. In the Vermont overfeeding study, cholesterol and triglycerides were increased and FFA reduced, although the values were not outside normal range [4,19]. In the long-term twin study where subjects were overfed by 1000 kcal/d for 84 out of 100 days, triglycerides increased, but cholesterol was unchanged, and HDL-cholesterol decreased raising the CHOL/HDL ratio by 20% [20]. In the present study, HDL-cholesterol and free fatty acids had a parabolic relationship with both low and high protein diets producing higher levels of HDL-cholesterol and plasma free fatty acids than in the individuals who ate the NPD diet.

Although not the primary focus of this paper, glucose, insulin, leptin, and both systolic and diastolic blood pressure were significantly increased by overeating. These changes are consistent with the changes in the same variables published in other studies of overeating [5]. Glucose and insulin increased in most studies of overeating, but the magnitude of the response differed with the duration of overfeeding and the type of macronutrients that were overfed [5]. When measured, hourly glucose and insulin rose during the nighttime of the first day of overeating, in contrast to free fatty acids which decreased [21]. Glucose and insulin increased in more than half of the 300 studies of overeating reviewed by Bray and Bouchard, and insulin was increased more often than glucose [5]. Leptin was increased in relation to the increase in body fat in this study and in the literature on overeating [5]. Both systolic and diastolic blood pressure increased in the present study and in some [22,23] but not in all studies [24,25]. Two studies of ambulatory blood pressure during overeating show that the variability in systolic BP, but no diastolic BP, increased significantly [22,26]. The response of BP during overeating was reduced in individuals with high cardiorespiratory fitness [24], and in those labeled as having “metabolically normal obesity” compared to individuals labeled as having “metabolically abnormal obesity” [27].

### 5.2. Overfed Protein

Data on overeating using low protein diets with 2 to 7% protein have been presented in 3 reports [6,12,13] which examined efficiency of weight gain but did not provide data on body composition or circulating lipids. In the present study, focusing on changes in lipids and liver fat, we showed that protein intake at the lower limits of recommended allowances [28] was associated with increased HDL-cholesterol, free fatty acids, and liver fat (i.e., reduced liver density) but reduced fat free mass (Figure 1). As noted above, however, individuals eating the low protein diet had the highest fat intake, and the analysis of the contribution of fat and protein to these changes showed that it was primarily dietary fat, especially saturated fat intake, that correlated best with the change in HDL-cholesterol, free fatty acids, and liver fat.

### 5.3. Overfed Fat

In the present study, there were measurements of total fat intake, as well as the amount of saturated fat, monounsaturated fat, and polyunsaturated fat in each of the diets which was determined chemically in composites of the three diets. Because of the differences in body weight among individuals in each dietary protein group, there was a range of fat intake in the LPD, NPD, and HPD groups. For example, in the LPD group, all 8 participants had >150 g/d of total fat, whereas 6 of the 9 participants in the NPD group and only 1 of the 6 in the HPD group consumed >150 g/d of fat/d. Individual fats showed different patterns of change in the variables shown in Table 3. Saturated fat, for instance, was associated with significant changes in 4 of the metabolic variables in this study. In contrast, total fat and monounsaturated fats were associated with changes in 2 variables, and polyunsaturated fat with only 1. Higher intake of fat was associated with a significant increase in body fat (*p* = 0.033) and visceral adipose tissue (VAT) (*p* = 0.038). Increased intake of saturated fat was also associated with a significant decrease in fat free mass (*p* = 0.0093). Statistical modeling of the effects of dietary fat and protein intake on changes in fat free mass, free fatty acids, HDL-cholesterol, and liver fat showed that saturated fat was associated with the increase in HDL-cholesterol, plasma free fatty acids, and the increase in liver fat. In a study by Rosqvist et al. [29] which compared diets enriched with either saturated fats or polyunsaturated fats, overfeeding the saturated fat diet increased visceral adipose tissue and liver fat compared to overeating the PUFA rich diet. Rosqvist et al. [29] also found that saturated fat increased visceral adipose tissue (VAT) which would be consistent with our observation that VAT was increased in relation to total fat, although not to saturated fat.

An increase in liver fat with overfeeding has been observed in several studies [23,29,30]. In a 56 day study, overeating increased intrahepatic lipid but not intramuscular lipid [23], and suggests that increased liver fat may be more detrimental than other forms of ectopic fat [31]. Another 56-day overfeeding study also showed an increase in intrahepatic lipid which rose from 16 ± 2% to 18 ± 2% [30]. Dietary fat and protein interact in the modulation of liver fat [32,33], indicating the need to include both of them when interpreting the data.

This study has several strengths. First, subjects were randomly assigned to one of 3 diets in a parallel arm study and remained on this diet and under observation while they overate their assigned diet for 8 weeks. Second diet composition was calculated from nutrient data bases and then composited diets were analyzed chemically, providing additional strength to the data on dietary fat. In addition, many preplanned sets of data were collected. There are also weaknesses in this study, including the small number of subjects in each group, and the fact that the analysis of the response to dietary fat was not included in the original analysis plan.

In conclusion, these studies show that the association of dietary protein with changes in plasma lipids during overeating are largely attributable to the reciprocal changes in dietary fat, especially saturated fat. The principal effect of dietary protein was on the changes in fat free mass. The other clear message is that higher levels of saturated fat increase HDL-cholesterol, plasma free fatty acids, and liver fat.

## Figures and Tables

**Table 1:** Diet composition during overeating.

Variable	LPD	NPD	HPD	*p* by ANOVA
Number of Participants (M/F)	8 (5M/3F)	9 (6M/3F)	6 (3M/3F)	
Age (years)	22.9 + 0.97	22.9 + 1.83	27.2 + 0.87	0.043
Weight (kg)	68.6 + 4.73	79.2 + 5.47	75.8 + 6.28	0.39
Calories fed (kcal/d)	3130 ± 129 *	3508 ± 228	3501 ± 309	0.14
Percent overfed	38 ± 3%	42 ± 3%	39 ± 5%	0.15
Carbohydrate Intake (g/d)(%)	341 ± 13 (39%)	369 ± 24 (46%)	378 ± 38 (45%)	0.57
Protein Intake (g/d)(%)	46.6 ± 1.66 (5%)	140.0 ± 9.62 (14%)	231.5 ± 23.0 (25%)	<0.0001
Total Fat (g/d)(%)	186 ± 7.1 (56%)	170.9 ± 10.8 (39%)	132 ± 11.1 (29%)	0.0054
Saturated Fat (g/d)	63.9 ± 3.0	44.6 ± 2.62	27.5 ± 2.49	<0.0001
Monounsaturated fat (g/d)	48.9 ± 2.1	45.8 ± 3.13	27.8 ± 2.56	<0.0001
Polyunsaturated fat (g/d)	30.4 ± 0.80	30.0 ± 1.76	15.3 ± 1.52	<0.0001

* Data are mean ± SEM; LPD = low protein diet (5% protein); NPD = normal protein diet (15% protein); HPD = igh protein diet (25% protein); M = male; F = female; ANOVA = Analysis of variance.

**Table 2:** Baseline and change from baseline by diet for the metabolic variable.

Variable	LPD		NPD		HPD		*p* for ANOVA	Overall *p* Value
	Baseline	Change	Baseline	Change	Baseline	Change		
Weight (kg)	68.6 ± 4.7	3.17 ± 0.46c	79.2 ± 5.95	6.26 ± 0.46c	75.8 ± 6.3	7.0 ± 0.49d	**=0.0028**	**<0.0001**
Body Fat (kg)	16.6 ± 1.8	3.65 ± 0.32d	18.3 ± 2.5	3.45 ± 0.50b	21.2 ± 2.7	3.62 ± 0.34d	=0.86	**<0.0001**
FFM (kg)	53.2 ± 3.65	−0.70 ± 0.24	59.9 ± 4.2	2.86 ± 0.47b	56.2 ± 64	3.24 ± 0.12c	**<0.0001**	**0.0011**
VAT (kg)	1.56 ± 0.31	0.51 ± 0.088b	1.58 ± 0.32	0.39 ± 0.11a	2.6 ± 0.63	0.47 ± 0.11c	=0.63	**0.0091**
DSAT (kg)	1.31 ± 0.49	0.63 ± 0.21a	1.31 ± 0.56	0.59 ± 0.21a	1.8 ± 0.61	0.72 ± 0.16b	=0.90	**<0.0001**
SSAT (kg)	4.21 ± 0.75	1.31 ± 0.21c	4.21 ± 0.90	1.14 ± 0.095d	5.0 ± 0.85	1.3 ± 0.06d	=0.63	**<0.0001**
Fat Cell Size (µL)	0.59 ± 0.068	0.20 ± 0.10	0.61 ± 0.10	0.11 ± 0.073	0.64 ± 0.10	0.13 ± 0.06	=0.92	**0.0041**
Liver Density (HU)	68.2 ± 4.65	−6.02 ± 2.05c	66.4 ± 2.78	0.17 ± 1.24	64.7 ± 5.4	1.49 ± 1.37	**=0.010**	0.23
Chol (mg/dL)	161 ± 8.6	31.2 ± 6.5b	163 ± 10.7	1.28 ± 1.3	182 ± 7.8	13.1 ± 6.0	**=0.015**	**0.0047**
Chol:HDL	3.83 ± 0.30	2.42 ± 2.29a	3.87 ± 0.28	7.54 ± 2.16a	3.71 ± 0.35	2.95 ± 2.65	=0.23	**0.0034**
HDL (mg/dL)	42.3 ± 2.2	15.1 ± 1.80d	43.7 ± 2.1	1.96 ± 1.56	50.4 ± 3.8	6.1 ± 1.8a	**<0.0001**	**<0.0001**
LDL (mg/dL)	94.6 ± 6.5	21.8 ± 6.3b	97.7 ± 8.6	1.83 ± 6.3	110.1 ± 6.5	13.6 ± 6.2	=0.088	**0.0068**
LDL:HDL	2.26 ± 0.21	1.77 ± 2.18	2.31 ± 0.20	2.28 ± 2.05	2.25 ± 0.24	3.55 ± 2.52	=0.86	0.16
TG (mg/dL)	118 ± 11.1	−28.9 ± 8.0	112 ± 25.7	−13.2 ± 6.8	106 ± 15.3	−33.5 ± 5.66b	=0.13	**<0.0001**
FFA (mmol/L)	0.29 ± 0.038	0.23 ± 0.11	0.32 ± 0.027	−0.027 ± 0.042	0.29 ± 0.06	0.025 ± 0.036	**=0.035**	0.13
Glucose (mg/dL)	85.2 ± 2.91	4.0 ± 1.1b	84.5 ± 2.64	4.44 ± 2.66	84.5 ± 1.2	4.8 ± 3.1	=0.97	**0.0030**
Insulin (µU/mL)	11.6 ± 1.7	1.6 ± 1.35	13.0 ± 3.02	1.24 ± 1.02	12.7 ± 2.43	1.15 ± 2.04	=0.59	**0.014**
Leptin (ng/dL)	11.6 ± 3.4	8.7 ± 2.3b	14.1 ± 4.7	5.9 ± 1.30b	20.1 ± 5.1	8.25 ± 1.65b	=0.50	**<0.0001**
SBP (mmHg)	103 ± 3.1	8.7 ± 2.3	106 ± 2.1	8.88 ± 3.5a	107 ± 2.3	12.1 ± 4.3a	=0.80	**=0.0002**
DBP (mmHg)	67 ± 2.6	4.25 ± 2.61	7.0 ± 2.1	2.48 ± 2.8	70 ± 3.5	4.0 ± 3.0	=0.88	**=0.036**

Data are Mean ± SEM; Statistical changes: a = *p* < 0.05; b = *p* < 0.01; c = *p* < 0.001; d = <0.0001. Abbreviations: FFM = Fat free mass; VAT = Visceral adipose tissue; DSAT = Deep subcutaneous adipose tissue; SSAT = Superficial subcutaneous adipose tissue; HDL = High density lipoprotein cholesterol; Chol = Cholesterol; LDL = Low-density lipoprotein cholesterol; TG = Triglyceride (triacylglycerol); FFA = Free fatty acids; SBP = Systolic blood pressure; DBP = Diastolic blood pressure.

**Table 3:** Linear correlation of total dietary fat, saturated fat, monounsaturated fat (MUFA), and polyunsaturated fat (PUFA) with changes in metabolic variables during overeating.

Variable	Total Fat		SFA		MUFA		PUFA	
	***r***	***p***	***r***	***p***	***r***	***p***	***r***	***p***
Weight (kg)	0.11	0.65	−0.11	0.13	0.00	0.97	−0.044	0.76
Body Fat (kg)	**0.49**	**0.033**	0.29	0.23	0.39	0.095	0.35	0.19
FFM (kg)	−0.39	0.61	**−0.58**	**0.0093**	−0.21	0.23	−0.24	0.18
VAT (kg)	**0.49**	**0.038**	0.34	0.17	0.38	0.12	0.24	0.31
DSAT (kg)	−0.024	0.93	−0.12	0.64	−0.086	0.75	0.11	0.67
SSAT (kg)	−0.058	0.79	−0.070	0.77	−0.13	0.61	−0.14	0.58
Fat Cell Size µL	0.096	0.84	0.14	0.65	0.050	0.92	−0.030	0.82
Liver Density (HU)	**−0.64**	**0.0046**	**−0.74**	**0.0008**	**−0.59**	**0.013**	**−0.49**	**0.045**
Cholesterol (mg/dL)	0.25	0.25	0.36	0.090	0.18	0.40	0.14	0.52
HDL:LDL	−0.091	0.68	−0.02	0.93	−0.045	0.84	0.014	0.95
HDL (mg/dL)	0.31	0.16	**0.51**	**0.014**	0.24	0.28	0.18	0.40
LDL (mg/dL)	0.11	0.60	0.20	0.36	0.054	0.80	0.020	0.92
TG (mg/dL)	0.27	0.21	0.20	0.53	0.34	0.12	0.34	0.11
FFA (mmol/L)	**0.56**	**0.0056**	**0.62**	**0.0016**	**0.48**	**0.0184**	0.37	0.083
Glucose (mg/dL)	0.22	0.31	0.08	0.71	0.18	0.42	0.12	0.59
Insulin (µU/mL)	0.10	0.65	0.069	0.75	0.14	0.54	0.94	0.67
Leptin (ng/mL)	−0.37	0.078	−0.16	0.46	−0.34	0.11	−0.28	0.18
SBP (mmHg)	0.016	0.81	−0.026	0.90	0.006	0.99	−0.05	0.82
DBP (mmHg)	0.35	0.10	0.20	0.35	0.27	0.22	0.23	0.29

Data are linear correlation coefficients and statistical significance thereof; Abbreviations: FFM = Fat free mass; VAT = Visceral adipose tissue; DSAT = Deep subcutaneous adipose tissue; SSAT = Superficial subcutaneous adipose tissue; HDL = High density lipoprotein cholesterol; Chol = Cholesterol; LDL = Low-density lipoprotein cholesterol; TG = Triglyceride (triacylglycerol); FFA = Free fatty acids; SBP = Systolic blood pressure; DBP = Diastolic blood pressure.
